# The impact of table tennis intervention on short video addiction among college students in China: the chained mediation effect of cognitive bias and self-control

**DOI:** 10.3389/fpsyg.2025.1666895

**Published:** 2025-12-17

**Authors:** Kanglin Wang, Jian Sun, Jiangtao Han, Zaihao Wu

**Affiliations:** 1 Xihua University, Chengdu, China; 2 Wuhan University of Technology, Wuhan, Hubei, China

**Keywords:** sports psychology, table tennis intervention, short video addiction, cognitive bias, self-control, the chained mediation effect

## Abstract

**Background:**

This study focuses on the impact of a 16-week table tennis exercise intervention on college students’ short video addiction and explores the chain mediating mechanism of cognitive bias and self-control therein. Currently, the short video addiction rate among college students is high, and physical exercise has shown unique value in digital addiction intervention. Still, the cognitive-behavioral level of the action path is not yet clear.

**Methods:**

Sixty college students with short video addiction (daily average usage duration ≥ 4 h) from a certain university in Chengdu, China, were selected and randomly divided into an experimental group (30 people) and a control group (30 people). The experimental group implemented a 16-week, 3-times-a-week moderate-intensity table tennis intervention (60–80% of the maximum heart rate), while the control group maintained regular activities. The Short Video Addiction Scale for College Students (SVAS), Negative Cognitive Processing Bias Questionnaire, and Self-Control Scale were used for pre- and post-tests. Repeated-measures analysis of variance and the Hayes Process plugin (Model 6) were used to test the mediating effect.

**Results:**

The table tennis intervention significantly reduced the short video addiction level and cognitive bias and improved self-control ability in the experimental group. The improvement was significantly better than that in the control group. Cognitive bias and self-control formed a chain mediating path, and the total indirect effect accounted for 68.33% of the total effect.

**Conclusion:**

Table tennis exercise can inhibit college students’ short video addiction through the chain mediating mechanism of reducing cognitive bias and enhancing self-control, providing an empirical basis from the perspective of sports psychology for digital addiction intervention.

## 1 Introduction

As of December 2024, the number of online video users in China reached 1.07 billion, among which 1.04 billion were short video users, accounting for 93.8% of the total netizens (China Internet Network Information Center [CINIC], 2025). A survey on young college students revealed that the detection rate of short video addiction among this group was as high as one-third (Ai et al., 2023). Short video addiction (SVA) refers to the phenomenon where individuals experience significant impairment in both physical and mental health due to excessive use of short videos. It is typically manifested as an inability to control the use of short videos, a decreased perception of time passing during video consumption, and difficulty in shifting attention to engage in other activities (Xue et al., 2025). Previous research has shown that SVA can severely damage physical and mental health. Excessive exposure to short videos can lead to a decline in daily performance indicators such as sleep quality and academic performance (Jiang and Yoo, 2024; Ye et al., 2022). Additionally, it can trigger negative psychological states, including depressive symptoms and a sense of meaninglessness in life (Wang et al., 2025).

Physical exercise has been proven to be an important intervention measure to alleviate short video addiction. Existing research indicates that appropriate participation in physical exercise can enhance the sensitivity of dopamine receptors, reduce excessive dependence on the immediate rewards brought by short videos, and restore the balance of the reward system (Lin and Kuo, 2013). At the executive control level, the recurrence of addictive behaviors can be significantly inhibited through physical exercise. A 12-week aerobic exercise intervention can effectively enhance the inhibitory control function of addicts, thereby substantially reducing the rate of behavioral recurrence (Ma et al., 2024; Yang et al., 2021). Notably, the effectiveness of physical exercise as an intervention has been verified in the digital addiction domains, such as smartphone addiction (Liu et al., 2019), addictive social media use (Brailovskaia and Margraf, 2020; Wang J. et al., 2024), and excessive online game use (Hong et al., 2020). Its action pathways and intervention logic possess theoretical transfer value for short video addiction. Based on the above cross-domain research evidence and specific mechanisms, this study posits the hypothesis that the level of participation in physical exercise can significantly and negatively predict the degree of short video addiction among college students.

Cognitive bias (CB) refers to a selective psychological tendency or deviation that occurs during the information-processing stage (Wilke and Mata, 2012). It leads individuals to prioritize the processing of information closely related to their traits, often resulting in errors and deviations from predetermined standards. According to Davis’s (2001) cognitive-behavioral model of internet addiction, non-adaptive cognition caused by negative evaluations is the primary cause of internet addiction (Yang et al., 2021). Existing research has demonstrated that CB directly affects smartphone addiction (Pang and Wu, 2023), loneliness, and social support, thereby influencing short video addiction. Moderate participation in physical exercise can effectively improve cognitive abilities (Fernandes et al., 2018). A randomized controlled experiment showed that engaging in sports can significantly reduce CB among healthy adults (Clarke et al., 2018). Moreover, moderate-intensity exercise has a more pronounced effect on improving CB (Cooper and Tomporowski, 2017). Based on these findings, this study hypothesizes that CB plays a mediating role between physical exercise and short video addiction among college students.

Self-Control (SC) refers to the ability of individuals to regulate their behaviors to meet social expectations and achieve personal values. Previous research has demonstrated that college students with high levels of self-control exhibit lower levels of smartphone addiction (Ding et al., 2022). In the face of the rich content of short videos and the extensive usage scenarios, individuals with strong self-control can browse short video content more rationally, and this conclusion also holds for the adolescent group (Fabio et al., 2022). Similarly, there is a significant negative correlation between self-control and problematic social media use (Świątek et al., 2023). Self-control increases individuals’ time-management tendencies, helping them establish a good sense of time in daily life and thus enabling adolescents to use their mobile phones more appropriately (Błachnio et al., 2023). Empirical studies have shown that individuals who engage in physical exercise experience a significant improvement in self-control (Wei, 2023). Additionally, a targeted intervention experiment has confirmed that aerobic exercise significantly enhances self-control (Oaten and Cheng, 2006). Based on the above, this study hypothesizes that self-control plays a mediating role between physical exercise and short video addiction among college students.

From the dual perspectives of cognitive neuroscience and mental resource theory, there is a close internal relationship between cognitive biases and self-control. The executive function of the prefrontal cortex, as the core neural basis, jointly supports the self-control process and the regulatory mechanism of cognitive biases (Hu et al., 2015). The limited resource theory of self-control provides a key framework for analyzing the interaction between the two. When cognitive biases are activated, individuals need to consume additional mental energy to correct these biases. This continuous depletion of resources can lead to a decline in the effectiveness of cognitive regulation, thereby weakening the executive validity of self-control ability. Existing research shows that the level of self-control significantly affects problem-based short video usage behavior by acting on an individual’s information-processing mechanism (Zhu and Fong, 2025). In the research on the neural mechanisms of gambling impulses, it has also been found that individuals with low self-control ability often exhibit more significant cognitive biases. The two form a vicious cycle of “resource depletion-bias reinforcement” during the process of behavioral regulation (Shi and Li, 2023). Based on this, this study hypothesizes that cognitive bias and self-control play a chain-mediated role between physical exercise and college students’ short video addiction.

As a sport with high cognitive demands, table tennis has been proven to have unique advantages in enhancing behavioral motivation and cognitive control (Zheng et al., 2024). Both the collaborative strategies in doubles scenarios and the competitive confrontations in singles can activate individuals’ need for belonging and social connections. This real-life social experience can replace the dependence on virtual interactions in short videos, thus alleviating the dependence on electronic products (Vansteene et al., 2022). Previous studies have mostly focused on the cross-section to explore the relationship between physical activity and electronic device addiction (Cheng et al., 2022). They are unable to distinguish the forms of physical activity, ignoring the unique effects of specific types of physical activity. Moreover, short video apps are different from traditional online games and mobile phones. Therefore, this study selects table tennis as the intervention activity for the experiment, explores the impact of physical exercise on short video addiction, and tests the mediating roles of cognitive bias and self-control in this process. This research method has been proven multiple times (Haoran et al., 2023; Wei et al., 2024).

**FIGURE 1:**
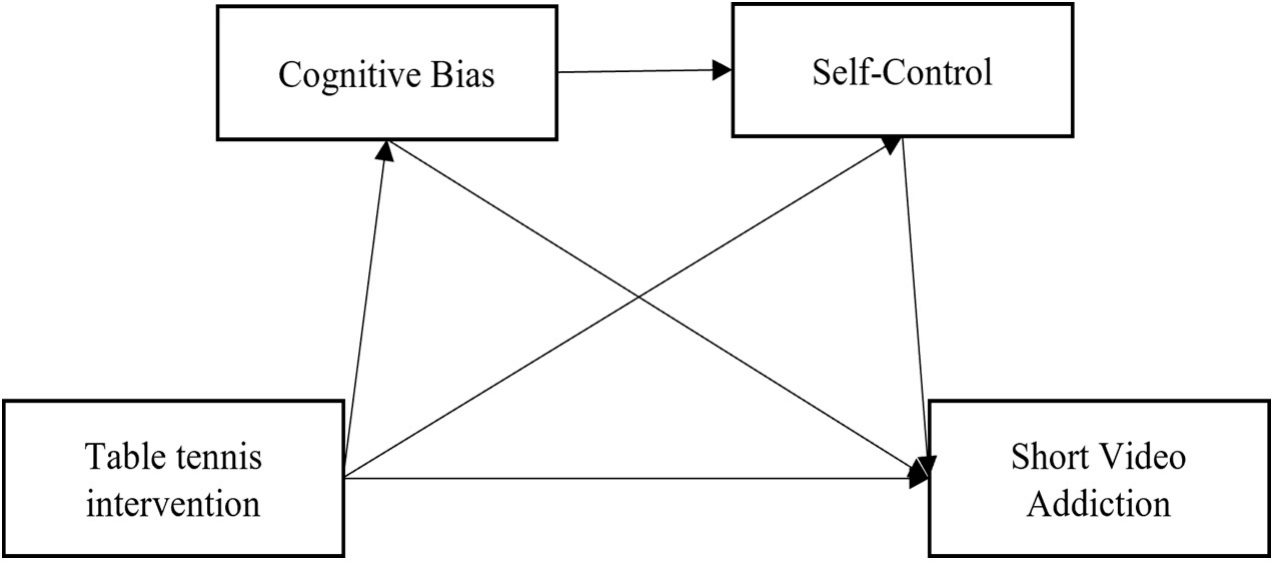
Model hypothesis diagram.

Based on the theoretical framework and literature review above, the present study establishes a chained mediation model (as depicted in Figure 1) and proposes the following specific research hypotheses: H1: Table tennis intervention will significantly reduce the level of short video addiction among college students. H2: Cognitive bias mediates the relationship between table tennis intervention and short video addiction. H3: Self-control mediates the relationship between table tennis intervention and short video addiction. H4: Cognitive bias and self-control play a chained mediating role in the relationship between table tennis intervention and short video addiction.

## 2 Materials and methods

### 2.1 Sample

Power analysis using G*Power 3.1 determined a minimum sample size of 54 participants (α = 0.05, power = 0.95, *d* = 0.80) (Faul et al., 2009), with 60 ultimately recruited to accommodate a 15% anticipated attrition rate (Brailovskaia and Margraf, 2020). By using the Short Video Addiction Scale (SVAS) in combination with the usage reports of short video applications on smartphones, a total of 63 college students who were addicted to short videos were recruited as participants from a university in Chengdu. Three students dropped out due to academic reasons before the start of the experiment, and finally, 60 students completed the entire experimental process. There are 7 diagnostic questions in the SVAS. If a participant gives affirmative answers to any 5 of these questions, they can be diagnosed as a severe short video addict, with a daily average usage duration of ≥ 4 h. All participants were randomly divided into an experimental group (17 males, 13 females) and a control group (15 males, 15 females), with an average age of 19.31 ± 1.36 years. This study strictly adheres to the ethical standards of the Declaration of Helsinki. All college students participating in this study have been informed of the research purpose, process, and potential risks, and have voluntarily signed a written informed consent form. This research has been approved by the Ethics Committee of Xihua University(XH250124-01).

### 2.2 Research tools

#### 2.2.1 International Physical Activity Questionnaire-Short

This scale was developed by the International Physical Activity Measurement Working Group in 2001 and has undergone reliability and validity tests in many countries and regions (Bosdriesz et al., 2012). The calculation method is that the physical activity score is the sum of vigorous physical activity, moderate physical activity, and walking activity.

#### 2.2.2 College students Short Video Addiction Scale

The measurement was carried out using the College Students’ Short Video Addiction Scale, developed by Qin (2020). It consists of four dimensions: Withdrawal, escapism, loss of control, and inefficiency, with a total of 14 items. This scale has been tested for reliability and validity in the college student population (Ding et al., 2024; Zhao and Kou, 2024). A 5-point Likert scale was used for scoring. The higher the total score, the more serious the individual’s short video addiction. In this study, the Cronbach α coefficient was 0.86. The results of the confirmatory factor analysis (CFA) in this study showed that: χ^2^*/*df = 2.85, RMSEA = 0.07, CFI = 0.92, TLI = 0.91.

#### 2.2.3 Cognitive Bias Scale

In this study, the “Negative Cognitive Processing Bias Questionnaire” developed by Yan et al. (2017). It includes dimensions of negative attention bias, negative memory bias, negative interpretation bias, and negative rumination bias, with a total of 24 items. A 5-point Likert scale was used for scoring. The higher the total score, the more serious the cognitive bias of the individual subjects. The Cronbach’s α coefficient in this study was 0.81. The results of confirmatory factor analysis showed that: χ^2^/*df* = 3.10, RMSEA = 0.08, CFI = 0.91, TLI = 0.90.

#### 2.2.4 Self-Control Scale

This study adopted the “Self-Control Scale” developed by Tangney et al. (2004), which includes five dimensions: Impulse control, healthy habits, resistance to temptation, focused work, and moderation in entertainment. There are a total of 19 items, among which 15 items use the reverse scoring method. A 5-point Likert scale is used for scoring. The higher the total score, the better the individual’s self-control ability of the subjects. This scale has been tested for reliability and validity by Wei (2023). The Cronbach’s α coefficient in this study is 0.84. The results of confirmatory factor analysis show that: χ^2^/*df* = 2.45, RMSEA = 0.06, CFI = 0.94, TLI = 0.93.

### 2.3 Experimental method

A 2 (Group: Experimental Group/Control Group) × 2 (Time: Pre-test/Post-test) mixed experimental design was utilized. The group served as the independent variable, while the SVAS score constituted the dependent variable, and cognitive bias and self-control functioned as the mediating variables. The experimental group was engaged with the table tennis project, whereas the control group, aside from regular physical activities, did not take part in any other forms of physical exercise.

### 2.4 Exercise intervention plan

**FIGURE 2:**
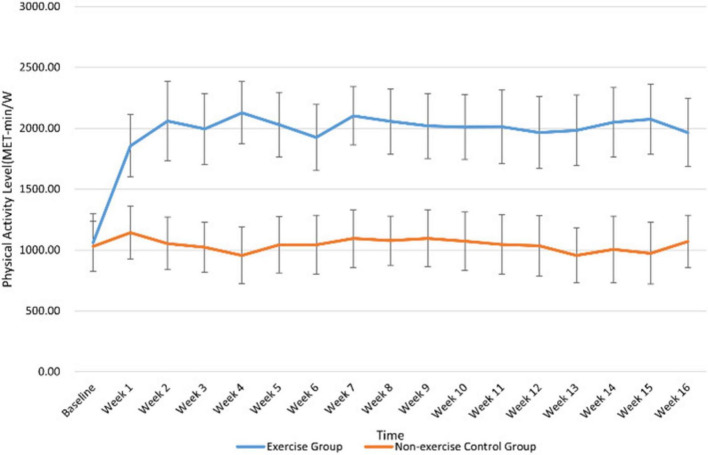
Dynamic changes in the physical activity levels of subjects in each group during the intervention.

The experimental group undergoes a 16-week table tennis exercise intervention three times a week (Pan et al., 2017; Tsai et al., 2017). Participants are required to, in addition to normal physical exercises, begin practicing a 60-min table tennis session (10-min warm-up + 40-min formal training + 10-min relaxation) at 7:00 p.m. every Wednesday, Friday, and Saturday. Before each experiment, five participants are randomly selected to wear Polar watches to monitor their heart rates, with exercise intensity controlled at a moderate level (60–80% of the maximum heart rate, approximately 110–150 beats per minute) (Sperlich et al., 2011). The single-training module includes dynamic stretching in the warm-up stage, racket-holding swing practice, technical training (30 min) that encompasses basic movement strengthening, footwork training, multi-ball continuous practice, and a physical fitness module (15 min) that consists of special physical fitness cycles, core stability training, and relaxation and recovery through static stretching. Record the average weekly physical activity level of the subjects through the IPAQ-S. Conduct a *t*-test on the physical activity levels of the two groups before the formal start of the intervention (*p* = 0.61 > 0.05), demonstrating that there is no significant difference in physical activity between the two groups before the start. After the formal experiment begins, the physical activity level of the exercise group increases significantly [(2025.13 ± 279.18) MET-min/w], while the physical activity level of the control group remains stable [(1055.63 ± 235.96) MET-min/w] (for details, see Figure 2).

### 2.5 Statistical analysis

This study used SPSS 25.0 and AMOS for statistical analysis, recoded the reverse-scored questions, applied the analysis of variance method, conducted correlation analysis, and collinearity tests on the data. Subsequently, the SPSS add-in PROCESS add-in developed by Hayes (2013) was used to test the mediating model. A significance level of *p* < 0.05 was set for statistical significance.

## 3 Research results

### 3.1 Preliminary analyses

#### 3.1.1 Common method bias analysis

Since the data in this study were obtained from the self-reports of the participants, they may be affected by common method bias. To ensure the normal progress of subsequent data analysis, before, the Harman single-factor test method was used to test for common method bias. The results showed that there were a total of 14 factors with eigenvalues > 1, and the explanatory rate of the first factor was 20.07% ( < the critical value of 40%) (Fuller et al., 2016). Thus, it can be seen that there is no serious common method bias in this study.

#### 3.1.2 Correlation analysis

Conduct a Pearson correlation analysis on cognitive bias, self-control, and short video addiction (post-test) (Table 1). The results show that there is a significant positive correlation between cognitive bias and short video addiction, and a significant negative correlation between cognitive bias and self-control; there is a significant negative correlation between self-control and short video addiction.

**TABLE 1:** Results of correlation analysis.

Variables	1	2	3	4
1 Table tennis intervention	1			
2 Cognitive bias	–0.65**	1		
3 Self-control	0.62**	–0.69*	1	
4 Short video addiction	–0.68**	0.71**	–0.74**	1

**p < 0.01,

*p < 0.05.

#### 3.1.3 Homogeneity test of different variables before the experiment

An independent samples *t*-test was used to compare the pre-test data of the experimental group and the control group. The results showed (Table 2) that there were no significant differences in short video addiction (SVA), cognitive bias (CB), and self-control (SC) between the two groups (*p* > 0.05), indicating that the variables were homogeneous before the experiment and met the conditions for subsequent analysis.

**TABLE 2:** Independent samples t-test of each variable before the experiment.

Variables	Group	*M*	*SD*	*t*	*p*	Cohen’s *d*
SVA	Experimental group	45.23	4.87	0.62	0.705	0.10
	Control group	44.87	5.12			
CB	Experimental group	79.83	9.76	–0.17	0.678	0.11
	Control group	80.27	10.34			
SC	Experimental group	49.67	7.92	–0.56	0.761	–0.08
	Control group	50.13	8.15			

### 3.2 The effects of table tennis on college students’ short video addiction, cognitive bias, and self-control

**FIGURE 3:**
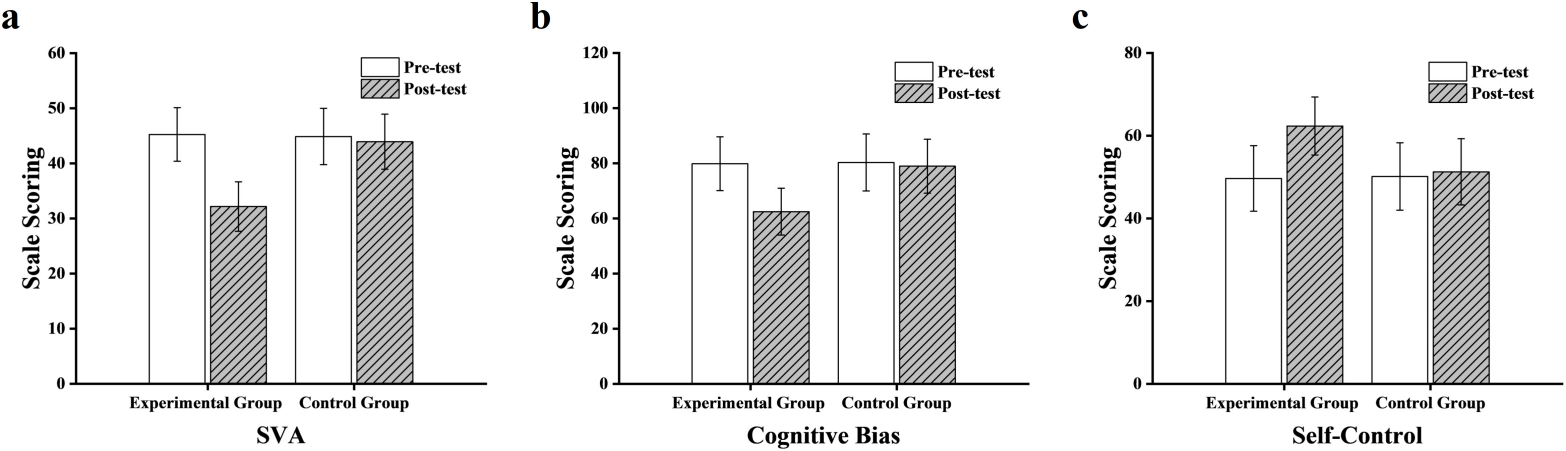
Simple effects analysis of the table tennis intervention on **(a)** short video addiction, **(b)** cognitive bias, and **(c)** self-control.

Repeated measures analysis of variance (Table 3) showed that there were significant differences in the main effect of time for short video addiction [*F*_(1, 58)_ = 85.24, *p* < 0.01, η_*p*_^2^ = 0.60], cognitive bias [*F*_(1, 58)_ = 92.17, *p* < 0.01, η_*p*_^2^ = 0.61], and self-control [*F*_(1, 58)_ = 78.93, *p* < 0.01, η_*p*_^2^ = 0.58], indicating that after the experiment, each variable showed a significant trend of change over time. In terms of the group effect, short video addiction [*F*_(1, 58)_ = 21.35, *p* < 0.01, η_*p*_^2^ = 0.27], cognitive bias [*F*_(1, 58)_ = 28.74, *p* < 0.05, η_*p*_^2^ = 0.33], and self-control [*F*_(1, 58)_ = 22.86, *p* < 0.01, η_*p*_^2^ = 0.28] in the experimental group were significantly better than those in the control group. In the interaction term of time × group, there were significant differences for short video addiction [*F*_(1, 58)_ = 64.82, *p* < 0.05, η_*p*_^2^ = 0.53], cognitive bias [*F*_(1, 58)_ = 76.53, *p* < 0.01, η_*p*_^2^ = 0.57], and self-control [*F*_(1, 58)_ = 61.12, *p* < 0.05, η_*p*_^2^ = 0.51]. Further simple effect analysis was carried out. In the experimental group, the post-test scores of short video addiction [*t*_(29)_ = 12.37, *p* < 0.001], cognitive bias [*t*_(29)_ = 13.25, *p* < 0.001], and self-control [*t*_(29)_ = 11.84, *p* < 0.001] were significantly better than those of the pre-test. There was no significant difference between the pre-test and post-test in the control group (*p* > 0.05). In the post-test, the scores of short video addiction [*t*_(58)_ = 8.96, *p* < 0.001], cognitive bias [*t*_(58)_ = 9.24, *p* < 0.001], and self-control [*t*_(58)_ = 7.85, *p* < 0.001] in the experimental group were significantly better than those in the control group (Figure 3).

**TABLE 3:** Analysis of variance of table tennis movement for various variables.

Variables	Sources of variation	Class III sum of squares	Mean square	*F*	η_*p*_^2^
SVA	Time	1256.84	1256.84	85.24**	0.60
	Time × Group	984.73	984.73	64.82*	0.53
	Group	642.15	642.15	21.35**	0.27
CB	Time	3482.91	3482.91	92.17**	0.61
	Time × Group	2890.47	2890.47	76.53**	0.57
	Group	1865.32	1865.32	28.74*	0.33
SC	Time	2937.58	2937.58	78.93**	0.58
	Time × Group	2274.65	2274.65	61.12*	0.51
	Group	1528.43	1528.43	22.86**	0.28

**p < 0.01,

*p < 0.05.

### 3.3 Test of the chain mediating effect

Correlation analysis revealed significant associations among variables. To mitigate covariance interference, all predictor variables were standardized using z-score transformation before multicollinearity diagnostics. Results indicated tolerance values of 0.86 for table tennis intervention (*VIF* = 1.16), 0.35 for cognitive bias (*VIF* = 2.86), and 0.32 for self-control (*VIF* = 3.12). All tolerance values ranged from 0.32 to 0.86 (exceeding the > 0.1 threshold), and *VIF* values ranged from 1.16 to 3.12 (below the < 5 cutoff). Supplementary diagnostics confirmed a condition index of 18.7 (below the 30 threshold), minimum eigenvalue of 0.28 (above 0.01), and maximum variance proportion of 0.63 (below 0.90). Consistent with established multicollinearity standards (Cohen et al., 2003; O’brien, 2007), these results confirm the absence of significant multicollinearity, satisfying prerequisites for subsequent mediation analyses (Cohen et al., 2003).

**FIGURE 4:**
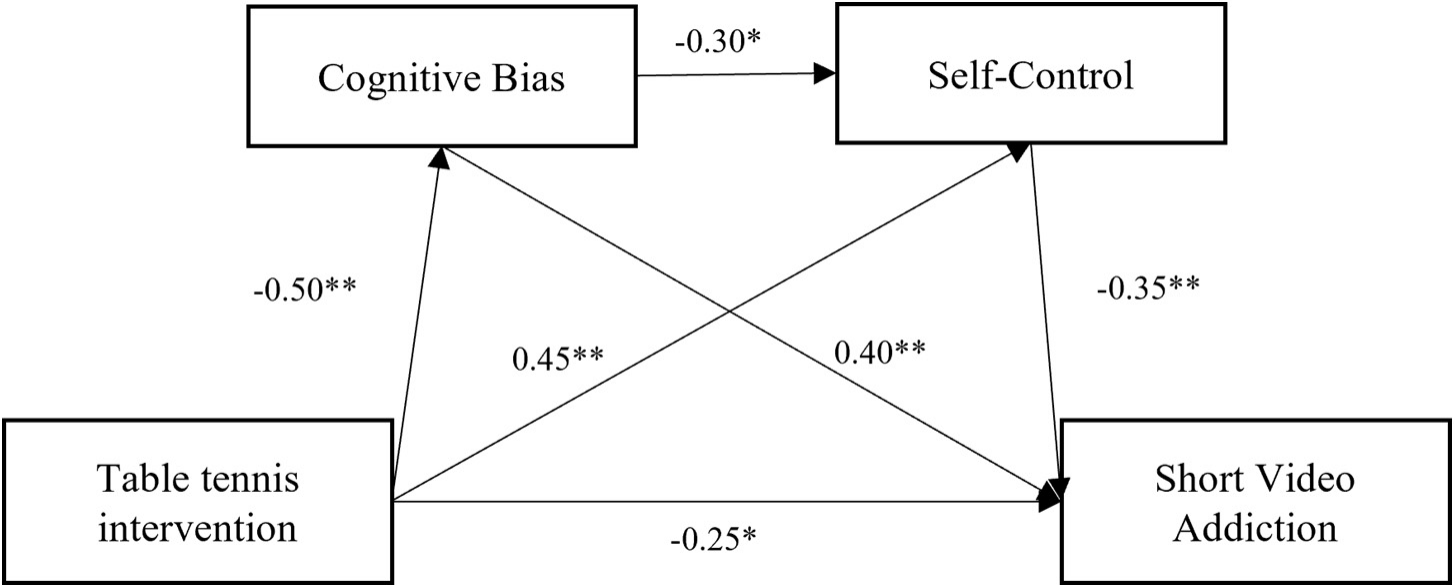
Path coefficient diagram. **p < 0.01, *p < 0.05.

Taking the group (exercise experimental group/control group) as the independent variable, short video addiction (change amount) as the dependent variable, and cognitive bias and self-control (change amount) as the mediating variables, the chained mediating effect test was conducted using the PROCESS plugin (Model 6, Bootstrap = 5,000) developed by Hayes. The results showed that table tennis exercise significantly and negatively predicted cognitive bias (*β* = –0.50, *p* < 0.01) and short video addiction (*β* = –0.25, *p* < 0.05), and significantly and positively predicted self-control (*β* = 0.40, *p* < 0.01); cognitive bias significantly and negatively predicted self-control (*β* = –0.30, *p* < 0.05), and significantly and positively predicted short video addiction (*β* = 0.40, *p* < 0.01); self-control significantly and negatively predicted short video addiction (*β* = –0.35, *p* < 0.01). For the results of regression analysis, please refer to Table 4; for the path model, please refer to Figure 4.

**TABLE 4:** Regression analysis of variables in the model.

Result variable	Predictive variables	*R*	*R* ^2^	*F*	β	*t*
Cognitive bias	Table tennis intervention	0.65	0.42	41.38*	–0.50	–6.25
Self-control	Cognitive bias	0.79	0.62	46.72**	–0.30	–3.33
	Table tennis intervention				0.45	6.43
Short video addiction	Self-control	0.85	0.72	28.15*	–0.35	–5.87
	Cognitive bias				0.40	6.67
	Table tennis intervention				–0.25	–2.78

**p < 0.01,

*p < 0.05.

The Bootstrap method was used to resample 5,000 times to calculate the 95% confidence interval. Table tennis can directly affect short video addiction, with a direct effect value of *β* = –0.25 [95% *CI* (–0.40, –0.10)]. Cognitive bias and self-control play a partial mediating role between table tennis and short video addiction, with a total mediating effect value of *β* = –0.41 [95% *CI* (–0.55, –0.27)], accounting for 68.33% of the total effect. Specifically, the mediating effect consists of the following three paths: Path 1: The indirect effect value is *β* = –0.20 [95% *CI* (–0.30, –0.10)], accounting for 33.3% of the total effect, indicating that table tennis indirectly reduces addiction by decreasing cognitive bias. Path 2 : The indirect effect value is *β* = –0.16 [95% *CI* (–0.25, –0.06)], accounting for 26.67% of the total effect, indicating that table tennis inhibits addictive behavior by enhancing self-control. Path 3 : The indirect effect value is *β* = –0.05 [95% *CI* (–0.10, –0.01)], accounting for 8.33% of the total effect, revealing the continuous mediating mechanism of cognitive bias and self-control (Table 5).

**TABLE 5:** Bootstrap analysis of the significance test of the mediating effect.

Effect type	β	Boot SE	95% CI	Proportion of relative effect
Direct effect	–0.25	0.09	[–0.40, –0.10]	41.67%
Total indirect effect	–0.41	0.07	[–0.55, –0.27]	68.33%
Indirect effect 1	–0.20	0.05	[–0.30, –0.10]	33.33%
Indirect effect 2	–0.16	0.05	[–0.25, –0.06]	26.67%
Indirect effect 3	–0.05	0.02	[–0.10, –0.01]	8.33%
Total effect	–0.60	0.07	[–0.74, –0.46]	100%

Indirect effect 1 = Table tennis→ Cognitive bias→ Short video addiction. Indirect effect 2 = Table tennis → Self-control → Short video addiction. Indirect effect 3 = Table tennis → Cognitive bias → Self-control → Short video addiction.

## 4 Discussion

### 4.1 Effect of table tennis intervention on college students’ short video addiction

Research shows that after 16 weeks of table tennis intervention, the short video addiction of college students has been significantly improved. Previous studies have indicated that physical exercise plays a positive role in alleviating mobile phone addiction and addictive social media use (Brailovskaia and Margraf, 2020; Zhao et al., 2022). The results of our experiment are consistent with these findings, demonstrating that a structured table tennis program can effectively reduce SVA scores. Specifically, the mechanism may be twofold. First, short video addiction is significantly associated with negative emotions such as depression and loneliness (Niu, 2023), and its essence is a virtual compensation in the absence of real-world social connections. Table tennis, as a team sport, creates high-frequency real-world social scenarios. It not only directly replenishes an individual’s psychological resources but also weakens the psychological dependence on virtual online social interactions by rebuilding real and intimate relationships, thus alleviating the psychological incentives for short video addiction at the root (Yang et al., 2025). Second, compared with traditional aerobic exercises, the skill training in racket sports (such as serving, spin control, and landing point prediction) can continuously stimulate an individual’s sense of challenge and provide immediate feedback. This engaging nature of table tennis may effectively compete for the time and attention that individuals would otherwise devote to short videos, thereby directly reducing usage time and dependency (Qian et al., 2025).

### 4.2 Effect of table tennis intervention on cognitive biases of college students

Research shows that after 16 weeks of table tennis intervention, the cognitive biases of college students have been significantly improved. This finding provides empirical support for Davis’s cognitive-behavioral theory model (Davis, 2001). This theory emphasizes the central role of maladaptive cognition in the mechanisms of addictive behavior and psychopathology. The results of this study reveal that table tennis can serve as an effective cognitive restructuring intervention. Longitudinal neuroimaging studies have shown that professional table tennis players are significantly superior to the general college student group in cognitive dimensions such as decision-making, visual attention regulation, and executive function. This is accompanied by an enhancement in the integrity of white matter fiber tracts in the brain and an improvement in neural conduction efficiency (Ji et al., 2024). The synergistic effect of this structural and functional change provides a biological basis for the improvement of cognitive biases (Padulo et al., 2016). Resting-state functional magnetic resonance imaging (fMRI) studies further confirm that table tennis training can optimize the functional connectivity networks in brain regions related to memory encoding, motor control, and visual information processing (Li et al., 2023). This aligns with existing research showing that sports participation can significantly reduce cognitive bias among healthy adults (Qi et al., 2024).

### 4.3 Effect of table tennis intervention on college students’ self-control

This study confirmed that after 16 weeks of structured table tennis intervention, the self-control ability of college students was significantly improved, and this finding is consistent with the existing research results (Liu et al., 2012). The high-frequency decision-making needs and dynamic attention allocation mechanism in table tennis continuously exercise an individual’s working memory and cognitive flexibility (Elferink-Gemser et al., 2018). When dealing with multi-source information, table tennis players have a faster cognitive switching speed than ordinary people. This improvement in cognitive flexibility helps individuals to carry out more efficient self-regulation when facing temptations (Wang and Qiu, 2025a; Wang Y. et al., 2024). In addition, as a typical team competitive sport, table tennis’s characteristics of competition and cooperation prompt participants to continuously adjust their behavioral strategies to adapt to environmental changes. While enhancing social cognitive abilities, it indirectly strengthens self-control abilities (Yu et al., 2022; Zhou et al., 2023). These findings, together with intervention studies on team sports such as basketball (Haoran et al., 2023; Wei et al., 2024), jointly reveal that dynamic interactive sports can effectively improve individuals’ self-control levels through the dual paths of cognitive training and social adaptation.

### 4.4 The chain mediating role of cognitive bias and self-control

This study found that cognitive bias and self-control play a significant chain-mediating role between table tennis exercise and short video addiction, thus supporting our hypothesis H4. The path analysis indicates that physical exercise reduces cognitive bias, which in turn enhances self-control ability, ultimately leading to reduced addiction. From the perspective of the limited resource theory, correcting cognitive bias requires consuming the limited psychological resources dominated by the prefrontal lobe. Continuous table tennis exercise systematically expands the reserve of psychological resources by enhancing the neural plasticity of the prefrontal cortex (Clarke et al., 2018). The results of this study are consistent with the conclusions of previous studies. Twelve weeks of moderate-intensity aerobic exercise significantly reduces the participants’ attentional bias toward addictive stimuli. At the same time, it enhances self-control ability by increasing the delay discount rate, directly verifying the effectiveness of the dual-mediation path (Cui and Yang, 2024; Yang et al., 2022). In addition, some research points out that in the digital media ecosystem, the algorithm recommendation mechanism reinforces addictive behaviors through immediate rewards (Ke et al., 2023; Kim et al., 2024; Wang and Qiu, 2025b). However, the cognitive restructuring triggered by physical exercise can weaken individuals’ neural adaptability to the immediate rewards of short videos. At the same time, the enhancement of self-control ability effectively blocks the positive feedback loop of addictive behaviors through the behavioral inhibition mechanism (Pindus et al., 2025). This dual mechanism reveals the unique value of physical exercise in the prevention and treatment of behavioral addiction in the digital age.

### 4.5 Practical and theoretical implications

This study offers several important implications. Practically, the findings provide a feasible and effective non-pharmacological intervention for universities to address the growing issue of short video addiction among students. Integrating structured racket sports, such as table tennis, into physical education curricula or extracurricular activities could serve as a preventative and remedial measure to enhance students’ mental wellbeing and self-regulation skills. Moreover, for mental health practitioners, the identified chain mediation pathway (cognitive bias → self-control) suggests that intervention programs could target cognitive restructuring and self-control training simultaneously for better efficacy. Theoretically, this research extends the cognitive-behavioral model of addiction by empirically validating it in the context of short video addiction and introducing physical exercise as a key antecedent. The demonstrated chain mediation effect enriches our understanding of the underlying psychological mechanisms, highlighting a sequential process where exercise first ameliorates maladaptive cognition, which in turn bolsters self-regulatory capacity, ultimately reducing addictive behaviors.

### 4.6 Research limitations and prospects

While this study provides evidence for the effectiveness of table tennis intervention, several limitations should be noted when interpreting the results, which also point to directions for future research. Firstly, the generalizability of the findings is limited by the sample, which was drawn from a single university. Future studies should recruit participants from multiple universities across different regions and cultural backgrounds to enhance the external validity of the findings. Secondly, the reliance on self-reported measures for key variables (e.g., short video addiction, self-control) might be influenced by social desirability. Incorporating objective measures, such as behavioral tasks for assessing cognitive bias and self-control, or using smartphone analytics to track actual usage time, could strengthen the validity of future research.

Thirdly, the design lacked an active control group. The observed effects might therefore be attributed to the extra social interaction and attention received by the experimental group, rather than the specific attributes of table tennis. Including an alternative exercise group in future designs would help isolate the unique effect of table tennis.

## 5 Conclusion

This study found that after a 16-week table tennis exercise intervention, college students’ short video addiction, cognitive bias, and self-control all improved. Table tennis exercise can influence short video addiction through cognitive bias and self-control. Meanwhile, cognitive bias and self-control play a chain-mediated role in the process of table tennis exercise, impacting short video addiction. Table tennis may be an effective intervention measure to address the addiction of college and middle school students to short videos, and it can be promoted within the school setting.

## Data Availability

The raw data supporting the conclusions of this article will be made available by the authors, without undue reservation.
